# Practical Observations on the Same

**Published:** 1840

**Authors:** M. Winans

**Affiliations:** Green county, Ohio


**Art. V.**—*Practical Observations on the Disease denominated Milk-Sickness or Sick-Stomach.* By Dr. M. Winans, of Green county, Ohio.

In the Western Journal of Medicine and Surgery, for January, 1840, I notice some remarks headed "Milk-Sickness *alias* Sick-Stomach." To these names I would add that of *Paralysis Intestinalis,* which expresses the nature of the disease more fully than any I have seen.

In this disease the great difficulty is to arouse the bowels to action ; the peristaltic motion is almost, and in some cases entirely, suspended, and yet the bowels are not usually constipated. I have rarely met with hardened fæces at any stage of the disease, and I may say that my practice has been pretty extensive. Formerly the disease prevailed every year in this section of country, (the eastern part of Green county, Ohio,) and I think it probable that I had from three to five hundred patients during the whole time the disease prevailed. It has now nearly or quite vanished away. I have met with but few cases for the last four or five years.

*Cause.*—This disease is evidently caused by a poison, which, I think, is of the vegetable class—because animals as well as men are subject to it in the sections of country where it prevails. But as to the particular vegetable which produces it, I am not fully satisfied. I am now of the opinion that it is produced by the *Champignon,* or at least by some of the mushroom tribe. This opinion is founded on the fact, that the disease never prevails except adjacent to thick, shady forests. When the woods are cleared off, the disease vanishes, and it never prevails in prairies or open ground of any kind. I was first led to this opinion by reading of the poisoning of the inhabitants of the West Indies by *Champignons* or mushrooms, which they used as food; but which, gathered from dense forests, were poisonous, although, when gathered in open grounds, they were eaten with impunity. I have noticed, in riding through our forests, a great variety of mushrooms of a small growth and of gaudy colors; some deep red, some pale red, some yellow, and some brown; and I have also observed them growing in beds of moss, of which animals are remarkably fond. I have, for many years, discarded the notion that the disease was produced by a weed of any kind. The hog is more subject to the disease than any other animal; and we know that he is very choice in his selection of weeds for food, and there are but few kinds that he will eat; but of the mushroom he is very fond.

*Symptoms.*—External nausea and vomiting, with a great loss of muscular power; very little, if any, fever or headache; skin cool and natural to the feel; great thirst; tongue clean or slightly furred; pulse not much excited; very little, if any, pain in any part of the body, but an uneasy, restless sensation, which is expressed by the eye, as well as by other motions of the body. These symptoms continue for four or five days, and then the vomiting ceases, but the nausea continues, and a torpor of the whole system supervenes; sometimes the coffee-grounds vomit precedes the termination in death.

### TREATMENT.

Calomel is the only medicine that we can expect the stomach to retain; and even it is frequently evacuated by the excessive vomiting. We have to resort to the syringe, and persevere day after day, until we overcome the paralyzed condition of the intestines. When we succeed in removing the disease, the convalescence is rapid, only requiring a few days for the re-appearance of good health.

*February,* 1840.

